# Preoperative factors analysis on root development after regenerative endodontic procedures: a retrospective study

**DOI:** 10.1186/s12903-022-02412-x

**Published:** 2022-09-04

**Authors:** Qian Zeng, Jianying Zhang, Jiang Guo, Shuya Liu, Maobin Yang, Jiacheng Lin

**Affiliations:** 1 Hospital of Stomatology, Guangdong Provincial Key Laboratory of Stomatology, Guanghua School of Stomatology, Sun Yat-sen University, Guangzhou, 510055 China; 2 Xiangya Stomatological Hospital, Xiangya School of Stomatology, Central South University, Hunan Key Laboratory of Oral Health Research, Changsha, Hunan China; 3 Department of Endodontology, Kornberg School of Dentistry, Temple University, Philadelphia, PA USA

**Keywords:** Regenerative endodontic procedures, Diagnosis, Preoperative factors, Root development

## Abstract

**Background:**

Regenerative endodontic procedures (REPs) have achieved clinical success on the immature permanent teeth with pulp necrosis, and can promote root development. However, preoperative factors and their effects on root development of REPs have not been definitely concluded. The aim of this study was to investigate the preoperative factors that may influence the root development of REPs.

**Methods:**

A total of 116 teeth in 110 patients treated with REPs in the Paediatric Dentistry Department and Endodontics Department from 2013 to 2017 were included in this study. Preoperative factors including aetiology, age, diagnosis and initial root morphology were collected retrospectively, and the associations between these factors and root development after REPs were analysed by Fisher's exact test and multivariate logistic regression model.

**Results:**

The overall rate of root development after REPs was 89.7%. The dens evaginatus group showed a higher rate (98.8%) in root development than the trauma group (67.6%) (*P* < 0.01). There was no significant difference among the different age groups (7–13 years old) or among different diagnoses groups (*P* > 0.05). And it showed in the trauma group that the teeth with apical foramen sizes larger than 3 mm significantly promoted root development than those smaller than 3 mm (*P* < 0.01). Multivariate logistic regression indicated that aetiology was significantly correlated with root development of REPs (OR: 0.07, 95% CI 0.007, 0.627, *P* < 0.05).

**Conclusions:**

The REPs promoted more root developments in the dens evaginatus group than the trauma group, indicating that aetiology may be correlated with the root development of REPs.

**Supplementary Information:**

The online version contains supplementary material available at 10.1186/s12903-022-02412-x.

## Background

Dental trauma, developmental malformation or caries in young permanent teeth can cause pulp necrosis and hinder root development. Regenerative endodontic procedures (REPs), a biologically based treatment, have been considered as effective modality for such nonvital immature teeth, aiming at continued root development as well as healing of apical lesion. Banchs and Trope introduced the modified clinical regenerative endodontic protocol in 2004 [1], and the recommendations of the American Association of Endodontists (AAE) for REPs have been revised several times due to the rapid advancement in research findings in this field. Nevertheless, the goals of REPs according to AAE consistently include the followings: primary goal (resolve symptoms and promote apical healing), secondary goal (root development), and tertiary goal (obtain a positive response to vitality testing) [2].

Numerous cases of REPs have achieved clinical success, which was defined as healing of periapical lesions and continued root development [3–6]. Several clinical studies have shown that the success rate of REPs was between 83.3 and 100% [7–10]. And failures in root development of teeth after REPs have also been reported [8, 11, 12]. Thus the prognostic factors that would influence the outcome of REPs have drawn dramatic attention. Up till now, the prognostic factors influencing the success rate of REPs have been reported in a few studies. It has been reported that age and the preoperative stage of root morphology, such as foramen diameter, could be associated with the success of the REPs [13–15]. In our previous study, a clinical randomized study was performed to compare the success rate between apexification and REPs, and the influence of etiology on the treatments was also explored [8]. The result showed that dens evaginatus cases had a higher success rate than trauma cases at 12 months among 69 inclusive cases receiving REPs [8], suggesting that the aetiology may also be a prognostic factor of REPs. However, up till now, no definite conclusion has been drawn about the influence of the above factors on the root development of REPs. The present retrospective study was performed on the basis of a relatively large sample size, aiming to analyse the preoperative factors (aetiology, age, diagnosis and initial root morphology) for the root change outcomes of REPs and assist decision making for treatment plan of immature permanent teeth with pulp necrosis.

## Methods

### Patient samples

Patients receiving REPs on immature permanent teeth with pulp necrosis or apical periodontitis from January 2013 to April 2017 were included. This study was approved by the Ethics Committee of the Affiliated Hospital of Stomatology, Sun Yat-sen University. All cases were treated at the Paediatric Dentistry Department and Endodontic Department of Hospital of Stomatology, Sun Yat-sen University, with at least 1 year follow-up. The treatments were performed by two dentists who had special training in paediatric dentistry and endodontics.

The REPs was performed according to the procedures described in a previous study [8]. Limited field of view cone beam computed tomography (FOV CBCT) (PHT-6500; VATECH Co., Ltd., Korea, 90 kV, 7.0 mA) was pre-operatively taken. 1.5% sodium hypochlorite (NaOCl) (Tanxiao Fenwei Pharmaceutical Co. LTD, China), and 17% EDTA (Zhongnan Reagent Industry Co. LTD, China) were used as irrigants during the treatment. Ciprofloxacin (Sigma Chemical Company, USA), metronidazole (Sigma Chemical Company, USA) and clindamycin hydrochloride (Sigma Chemical Company, USA) were mixed in 1:1:1 with sterile water and delivered into the canals as intracanal medicament. An absorbable collagen barrier (Heal-all Biological Membrane; Zhenghai Biological Technology Co. LTD, China) followed by WMTA (ProRoot white MTA; Dentsply International, Inc., Germany) was placed on the top of blood clot before the permanent restoration. The patients were recalled at 3, 6, 12, and 24 months and then yearly after treatment.

### Data collection

The following variables were retrospectively collected from patient records: (a) age when the REPs were initiated, (b) tooth type, (c) aetiology, (d) diagnosis, and (e) preoperative root morphology. The root morphology data from FOV CBCT taken before REPs and 1 year after REPs was analysed, and the root morphology data, including root length, root wall thickness and apical foramen diameter, were measured by one experienced oral radiologist using Ez3D2009 software according to a previous study [8]. Briefly, axial planes X, Y, and Z were used to determine the central location of the measurement. The X axial plane was parallel to the long axis of the teeth with Y axial plane perpendicular to the X axial plane and crossed the maximum diameter of the pulp from the mesio-distal direction. The Z axial plane was perpendicular to both the X and Y axial planes and connected the top of the alveolar ridge crest mesial and distal to the teeth. The distance between the cemento-enamel junction (CEJ) and the apical endpoint was measured distally, mesially, buccally and lingually, which were then averaged as the root length. The size of the apical foramen was averaged from the diameters of the buccolingual and mesiodistal directions. The root thickness was the average value of the thickness at 4 mm, 6 mm and 8 mm from the CEJ and from the bucco-lingual and mesio-distal directions (Additional file 1).

Root development was classified into four types according to the postoperative CBCT results at 1 year follow-up. Type I was defined as an increase in root length and a decrease in apical foramen. Type II and Type III were defined as only increases in root length or decreases in the apical foramen, respectively, while Type IV was regarded as unchange in root length and apical foramen [8]. Outcomes of Type I, Type II and Type III were regarded as continued root development after REPs.

The success of REPs was defined as elimination of symptoms and disappearance of apical radiolucency (AAE primary goal). The failure of REPs was defined as one of the followings: the presence of clinical symptoms (pain, swelling or sinus tract), root fracture or recurrence of apical periodontitis.

### Statistical analysis

All data were analysed using IBM SPSS 25.0 software, and a *P* value of 0.05 or less was considered to indicate statistical significance. Demographics and clinical data were expressed as percentages for categorical variables, means with standard deviations (SDs) and median for continuous variables.

For univariate analysis, Fisher's exact test was used for categorical variables. Besides, a multivariate logistic regression model was used to identify the preoperative factors influencing the root development of REPs. Gender, age, tooth type, aetiology, diagnosis and preoperative root morphology (apical foramen size, root length, and root canal wall thickness) were included in the regression model according to the professional and univariate outcomes using the type IV group as a reference. For the logistic regression model, the odds ratio (OR) and 95% confidence interval (95% CI) were used to describe the results. The Hosmer–Lemeshow test was used to evaluate the goodness of fit of the model.

## Results

From 2013 to 2017, 132 young permanent teeth in 126 patients were treated with REPs. By June 2019, 15 patients with 15 teeth (11 cases identified by paper records and 4 cases by electronic medical records) were lost within 1 year follow-up with a recall rate of 88.9%, and 1 tooth was extracted because of orthodontic requirements within 1 year after REPs. Therefore, a total of 116 teeth in 110 patients were included in this study. The included patients’ demographic and clinical details were shown in Additional files 2 and 3, and Table 1 summarized the demographic and clinical data of the study population. In present study, all premolar cases were caused by dens evaginatus and the incisor cases were caused by dental trauma. The age of the patients ranged from 7 to 13 years old. The average age of trauma group was 8.9 ± 0.5 years old and that of dens evaginatus group was 10.9 ± 0.8 years old.

**Table 1:** Demographics and clinical data of the study population

Variables	Categories	Value
Age (years)	Mean ± SD	10.3 ± 0.7
Gender, N (%)	Male	52 (47.3%)
	Female	58 (52.7%)
Tooth type, N (%)	Maxillary central incisors	32 (27.6%)
	Maxillary lateral incisors	2 (1.7%)
	Maxillary second premolars	3 (2.6%)
	Mandibular second premolars	76 (65.5%)
	Mandibular first premolars	3 (2.6%)
Aetiology, N (%)	Dental trauma	34 (29.3%)
	Dens evaginatus	82 (70.7%)
Diagnosis, N (%)	Asymptomatic apical periodontitis	77 (66.4%)
	Symptomatic apical periodontitis	18 (15.5%)
	Chronic apical abscess	16 (13.8%)
	Acute apical abscess	5 (4.3%)
Apical foramen size in trauma group (mm)	Median	3.00
Apical foramen size in dens evaginatus group (mm)	Median	2.65
Root length in trauma group (mm)	Median	12.9
Root length in dens evaginatus group (mm)	Median	11.50
Root thickness in trauma group (mm)	Median	1.41
Root thickness in dens evaginatus group (mm)	Median	1.41

Among all 116 cases, only 1 incisor case was retreated with apexification due to infection recurrence 3 months after REPs, and 115 cases achieved apical healing and were clinically asymptomatic, reaching a success rate of 99.1% by the primary goal of AAE at 1 year follow-up. 98, 41, 18 and 11 patients were followed-up for 2 years, 3 years, 4 years and 5 years, respectively, and did not present any symptom/sign of infection recurrence except that only 1 case caused by trauma showed up with a sinus tract at 2 years and 3 months after REPs and ended with extraction due to the recurrent infection.

Root development was analysed by comparing the CBCT images at the one year follow-up (Additional file 4) to the preoperative CBCT images. A total 104 out of 116 teeth achieved root development (Type I, II or III), with a rate of 89.7% by the secondary goal of AAE. The dens evaginatus group had Type I 86.6% (71/82 cases), Type II 9.8% (8/82 cases), Type III 2.4% (2/82 cases), and Type IV 1.2% (1/82 cases), while the trauma group had Type I 44.1% (15/34 cases), Type II 2.9% (1/34 cases), Type III 20.6% (7/34 cases) and Type IV 32.4% (11/34 cases). The statistical analysis showed a significant difference in the outcome distribution between the dens evaginatus group and trauma group (*P* < 0.001) (Table 2), indicating that aetiology may affect the root development of REPs.

**Table 2:** The influence of aetiology and diagnosis on root changes after REPs

Factors	Categories	Type I n = 86	Type II n = 9	Type III n = 9	Type IV n = 12	In total n = 116	*P*^△^
Aetiology	Dens evaginatus	71 (86.6%)	8 (9.8%)	2 (2.4%)	1 (1.2%)	82 (70.7%)	< 0.001***
	Trauma	15 (44.1%)	1 (2.9%)	7 (20.6%)	11 (32.4%)	34 (29.3%)	
Diagnosis	Symptomatic apical periodontitis	14 (77.8%)	0 (0.0%)	3 (16.7%)	1 (5.6%)	18 (15.5%)	0.608
	Asymptomatic apical periodontitis	56 (72.7%)	6 (7.8%)	6 (7.8%)	9 (11.7%)	77 (66.4%)	
	Acute apical abscess	4 (80.0%)	1 (20.0%)	0 (0.0%)	0 (0.0%)	5 (4.3%)	
	Chronic apical abscess	12 (75.0%)	2 (12.5%)	0 (0.0%)	2 (12.5%)	16 (13.8%)	

***P < 0.001, Δ Fisher's exact test

Next, whether diagnosis would influence the outcomes of REPs was evaluated. Table 2 showed that most cases achieved Type I outcomes despite different diagnoses, and overall, no significant difference was found among the four types of distribution of treatment outcomes based on the diagnosis (*P* > 0.05), suggesting that diagnosis may not influence root development of REPs.

The effect of age on REPs outcomes was also analysed. We first analysed the effect of each age subgroup on the outcome of REPs (7, 8, 9, 10, 11, 12, 13 years old) and found no significant difference among all age groups within each aetiology group. Next, the average ages of the dens evaginatus group and trauma group (10.9 and 8.9 years old, respectively) were used for statistical analysis to explore the effect of age on root development after REPs and there was no significant difference among them (≥ 10.9 years old. vs. < 10.9 years old in the dens evaginatus group and ≥ 8.9 years old vs. < 8.9 years old in the trauma group) (*P* > 0.05) (Table 3).

**Table 3:** The influence of age on root changes after REPs

Aetiology	Age	Type I n = 86	Type II n = 9	Type III n = 9	Type IV n = 12	In total n = 116	*P*^△^
Dens evaginatus	< 10.9	32 (84.2%)	4 (10.5%)	2 (5.3%)	0 (0.0%)	38 (46.3%)	0.413
	≥ 10.9	39 (88.6%)	4 (9.1%)	0 (0.0%)	1 (2.3%)	44 (53.7%)	
Trauma	< 8.9	8 (47.1%)	0 (0.0%)	3 (17.6%)	6 (35.3%)	17 (50.0%)	1.000
	≥ 8.9	7 (41.2%)	1 (5.9%)	4 (23.5%)	5 (29.4%)	17 (50.0%)	

Δ Fisher's exact test

Statistical analysis was performed to determine whether preoperative root morphology (apical foramen size, root length and thickness) affected outcomes of REPs. First, we analysed whether there was any difference in REPs outcomes among each range of apical foramen size (1, 2, 3 mm), root length (8, 9, 10, 11, 12 mm) and root wall thickness (1.2, 1.3, 1.4 mm) and found no significant difference among each range. Thus, the median values of these three indexes were calculated for statistical analysis. In the dens evaginatus group, preoperative root morphology did not affect the root development of REPs (*P* > 0.05) (Table 4). In the trauma group, only the teeth with apical foramen sizes larger than 3 mm achieved significant root development than those smaller than 3 mm (*P* < 0.01) (Table 4).

**Table 4:** The influence of preoperative root morphology on root changes after REPs

Aetiology	Root morphology	(mm)	Type I	Type II	Type III	Type IV	In total n = 116	*P*^△^
Dens evaginatus	Apical foramen size	< 2.65	31 (81.6%)	4 (10.5%)	2 (5.3%)	1 (2.6%)	38 (46.3%)	0.319
		≥ 2.65	40 (90.9%)	4 (9.1%)	0 (0.0%)	0 (0.0%)	44 (53.7%)	
	Root length	< 11.50	35 (87.5%)	5 (12.5%)	0 (0.0%)	0 (0.0%)	40 (48.8%)	0.486
		≥ 11.50	36 (85.7%)	3 (7.1%)	2 (4.8%)	1 (2.4%)	42 (51.2%)	
	Root thickness	< 1.41	39 (81.3%)	7 (14.6%)	1 (2.1%)	1 (2.1%)	48 (58.5%)	0.182
		≥ 1.41	32 (94.1%)	1 (2.9%)	1 (2.9%)	0 (0.0%)	34 (41.5%)	
Trauma	Apical foramen size	< 3.00	13 (52.0%)	1 (4.0%)	1 (1.0%)	10 (40.0%)	25 (73.5%)	0.001**
		≥ 3.00	2 (22.2%)	0 (0.0%)	6 (66.7%)	1 (11.1%)	9(26.5%)	
	Root length	< 12.90	8(42.1%)	1 (5.3%)	4 (21.1%)	6 (31.6%)	19 (55.9%)	1.000
		≥ 12.90	7 (46.7%)	0 (0.0%)	3 (20.0%)	5 (33.3%	15 (44.1%)	
	Root thickness	< 1.41	4 (40.0%)	0 (0.0%)	2 (20.0%)	4 (40.0%)	10 (29.4%)	0.921
		≥ 1.41	11 (45.8%)	1 (4.2%)	5 (20.8%)	7 (63.6%)	(70.6%)	

**P < 0.01,Δ Fisher's exact test

To further confirm the above results, multivariate logistic regression was also conducted, and the results demonstrated that aetiology was correlated with root development of REPs with a regression coefficient of − 2.687, OR 0.07, and 95% CI (0.007, 0.627) (*P* = 0.018) (Table 5).

**Table 5:** Multivariate analysis of root changes after REPs

Factors	Regression coefficient	Standard error	OR 95% CI	*P*Δ
Aetiology	− 2.687	1.133	0.07 (0.007, 0.627)	0.018*

*P < 0.05, Δ multivariate logistic regression

## Discussion

Endodontic treatment of immature permanent teeth with necrotic pulp consistently challenges clinicians due to the weak root wall and divergent apical foramen. To date, the REPs has been widely used to treat the above cases because they allow further increases in root length and root wall thickness, leading to the closure of apical foramen [6, 16–19]. Although the literature has demonstrated the efficacy of REPs in apical lesion healing and continued root development, failed REPs and absence of root development after REPs are also reported [11, 12, 20, 21]. A few studies have investigated the prognostic factors affecting the outcomes of REPs to guide clinical work [10, 13, 14]. However, no definite conclusion has been drawn due to the different study designs and the limitation of sample sizes (50, 62, and 46 cases receiving REPs, respectively) in these studies [10, 13, 14]. To understand the possible prognostic factors influencing the outcome of REPs, we designed this retrospective study based on our REPs database with a relatively large sample size of 116 patients from 2013 to 2017. As we know, preoperative factors and treatment protocols may affect the outcomes of REPs. In the present study, we focused on the influence of preoperative factors on the root development of REPs due to the standard operative procedures for the included patients.

The REPs have achieved favorable outcomes with high success rates ranging from 83.3 to 100% for immature teeth with apical periodontitis [7–10]. However, growing evidences showed that failed REPs could be found with persistent infection, root resorption and fracture [17, 22–24]. The systematic analysis by Almutairi et al. found that 79% of failed RET cases were presented with persistent infection, and 22 out of 37 failed cases (56%) were caused by dental trauma, and 39% of failed RET cases were identified after more than 2 years of follow-up [25]. In present study, two cases caused by dental trauma failed because of the infection recurrence within 1 year and over 2 years after REPs, respectively. The possible reason for the failure may be associated with the etiology of dental trauma, which may damage the blood supply in the apical area and decrease the resistance to infection. For the failed REPs, Lee and Song have raised that all endodontic procedures, second REP, apexification, conventional RCT, surgical approaches, and extraction could be considered modalities according to treatability of the tooth, accessibility to the canal, and the presence of an apical seat [24]. Apexification in nonvital permanent immature teeth with corono-radicular adhesive restoration can successfully achieve a favorable long-term outcome and may be a conservative alternative for the failed REPs [26]. In our study, apexification was chosen for the failed case within 1 year due to the absence of an apical seat and extraction for the failed case over 2 years because of the persistent infection and patient’s requirement.

Dens evaginatus, trauma and dental caries are the major causes of immature permanent teeth with necrotic pulp and apical periodontitis. A meta-analysis reported that there was no evidence of a difference in aetiology for the outcomes of REPs, in which success was defined as teeth being asymptomatic and teeth not requiring any other endodontic treatment after REPs (primary goal of AAE) [27]. However, our previous prospective study found that REPs cases with dens evaginatus had significantly better outcomes than those with an aetiology of dental trauma in achieving root development with a simple size of 69 REPs cases [8]. Chrepa et al. also reported that aetiology was a significant predictor of failure as well as root development [10]. In our present study, all the cases were caused by dens evaginatus or trauma, and the results confirmed that dens evaginatus cases showed a better prognosis than trauma cases in terms of root development with a relatively large sample size of 116 REPs cases, which was also verified by multivariate logistic regression. This may be because dental trauma induces damage to the apical papilla and Hertwig epithelial root sheath, which might lead to failure of continued root maturation. Within the limitation of the present study, we may conclude that aetiology would affect root development rather after REPs.

The diagnosis was evaluated as a potential prognostic factor in this study. We used current AAE diagnostic terminology and divided the cases into four clinical categories according to periapical status: asymptomatic apical periodontitis, symptomatic apical periodontitis, chronic apical abscess, and acute apical abscess [28]. The retrospective study by Chrepa et al. stated that apical diagnosis based on AAE criteria was considered a significant predictor for radiographic root area (RRA) change after REPs, indicating that the status of infection/inflammation at the apical area could influence the regulation of root development [10]. In contrast, our results showed that the diagnosis did not significantly affect root development after REPs defined by our study, suggesting that the clinical diagnosis may not be used as case selection for REPs. The opposite outcomes may be due to the different variables and statistical approaches used in the studies. In our opinion, regardless of the diagnosis, microbial control is the foundation for regenerative endodontic treatment, and appropriate disinfection of the canal is needed to achieve apical healing. Once the infection is well controlled, root development would be possible.

It has been reported that younger patients have a better healing ability in terms of dental pulp regeneration [10]. Estefan et al. explored the influence of age (9–18 years old) on the success of REPs and found that compared to the older age group (14–18 years old), the younger age group (9–13 years old) showed a significant increase in length independent of the preoperative size of apical diameter [14]. Chrepa et al. conducted a San Antonio study, in which patient ages ranged from 7 to 26 years, and found age was one of the significant predictors of failure and RRA change with an increase in age being associated with less gain in RRA [10]. However, our results showed no significant difference between ages on root development of REPs. This could be because the age range of patients in present study was between 7 and 13 years old with great healing ability and stem cell regenerative potential. Including samples with a wider age range (> 13 years old) should be considered for future studies to achieve more specific outcomes.

Preoperative root status/root morphology has also been evaluated as a prognostic factor of REPs. The study from Estefan et al. found that teeth with wider diameters (≥ 1 mm) demonstrated greater increases in root thickness, length, and apical narrowing [14]. Fang et al. conducted a literature search and concluded that teeth with apical diameters < 1.0 mm achieved clinical success after REPs, and teeth with apical diameters of 0.5–1.0 mm attained the highest clinical success rate [15]. In our study, the initial apical foramen size in the trauma group impacted the root development of REPs. The larger apical foramen achieved more Type I outcomes after REPs, which may be due to the abundant blood supply provided via the large apex.

## Conclusion

Within the limitation of the present study, we demonstrated that aetiology and initial apical foramen size in the trauma cases may correlate with root development after REPs. The REPs is proved to achieve more root development in the cases caused by dens evaginatus than in those caused by trauma based on a relatively large sample size. The present retrospective study provides additional evidence for the preoperative factors affecting the root development of REPs and may help clinicians make decisions when choosing the treatment plan for immature permanent teeth with necrotic pulps.

## Supplementary Information

**Additional file 1.** CBCT measurement of root length, apical foramen size and root thickness preoperatively and at the one year follow-up.

**Additional file 2.** Summary of patients’ demographic data.

**Additional file 3.** Preoperative measurement of root morphology by CBCT.

**Additional file 4.** Postoperative measurement of root morphology by CBCT at one year follow-up.

## Data Availability

The datasets analysed during the current study are available from the corresponding author on reasonable request.

## Abbreviations

REPs: Regenerative endodontic procedures
AAE: American Association of Endodontists
NaOCL: Sodium hypochlorite
CI: Confidence interval
FOV CBCT: Field of view cone beam computed tomography
SDs: Standard deviations
CEJ: Cemento-enamel junction
