# Supplementary material for: Preoperative factors analysis on root development after regenerative endodontic procedures: a retrospective study
Source: BMC Oral Health. 2022 Sep 4;22:374. doi: 10.1186/s12903-022-02412-x (PMC9442966; doi:10.1186/s12903-022-02412-x)
Supplement: Supplementary file 1 — Additional file 1. CBCT measurement of root length, apical foramen size and root thickness preoperatively and at the one year follow-up. [file 12903_2022_2412_MOESM1_ESM.pdf]

Supplementary 1. Figure legends

Figure 1 CBCT measurement of root length, apical foramen size and root thickness preoperatively (A-D) and at the 12-month follow-up (E-H). (A, C, E, G: Sagittal view. B, D, F, H: coronal view)

A, B, E, F: The distance between the CEJ and the apical endpoint was measured distally, mesially, buccally and lingually, which were then averaged as the root length. The size of the apical foramen was averaged from the diameters of the bucco-lingual and mesio-distal directions. (l: preoperative root length, L: root length at 1-year follow-up, w: preoperative apical foramen size, W: apical foramen size at 1-year follow-up).

C, D, G, H: The root thickness was the average value of the thickness at 4 mm (t4, T4), 6 mm (t6, T6) and 8 mm (t8, T8) from the CEJ and from the bucco-lingual and mesio-distal directions. (t: preoperative root thickness, T: root thickness at 1-year follow-up)

Figure

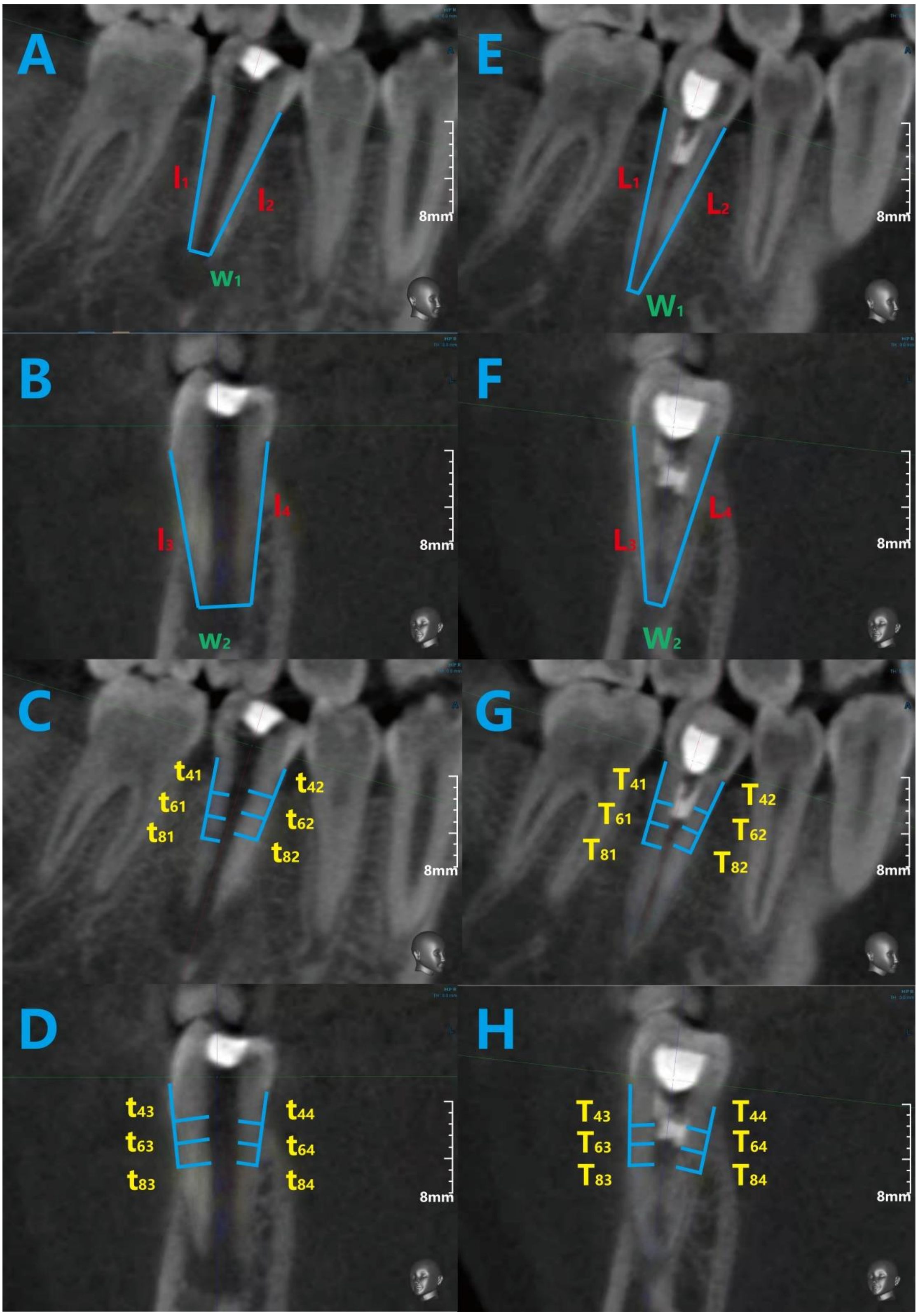
A
l1
l2
w1
8mm
E
L1
L2
W1
8mm
B
l3
l4
w2
8mm
F
L3
L4
W2
8mm
C
t41
t61
t81
t42
t62
t82
8mm
G
T41
T61
T81
T42
T62
T82
8mm
D
t43
t63
t83
t44
t64
t84
8mm
H
T43
T63
T83
T44
T64
T84
8mm
