# Supplementary material for: Preoperative factors analysis on root development after regenerative endodontic procedures: a retrospective study
Source: BMC Oral Health. 2022 Sep 4;22:374. doi: 10.1186/s12903-022-02412-x (PMC9442966; doi:10.1186/s12903-022-02412-x)
Supplement: Supplementary file 2 — Additional file 2. Summary of patients’ demographic data. [file 12903_2022_2412_MOESM2_ESM.docx]

|  |  | | | Table 1. Summary of Patients’ Demographic Data | | | |
| --- | --- | --- | --- | --- | --- | --- | --- |
| Patient no. | | Gender | Tooth position | | Age (y) | etiology | Pre-operative diagnosis |
| 1 | | male | 35 | | 14 | dens evaginatus | chronic apical abscess |
| 2 | | male | 45 | | 11 | dens evaginatus | asymptomatic apical periodontitis |
| 3 | | male | 35 | | 12 | dens evaginatus | asymptomatic apical periodontitis |
| 4 | | male | 35 | | 11 | dens evaginatus | asymptomatic apical periodontitis |
| 5 | | female | 35 | | 12 | dens evaginatus | symptomatic apical periodontitis |
| 6 | | female | 35 | | 13 | dens evaginatus | asymptomatic apical periodontitis |
| 7 | | male | 45 | | 14 | dens evaginatus | asymptomatic apical periodontitis |
| 8 | | female | 35 | | 10 | dens evaginatus | acute apical abscess |
| 9 | | male | 35 | | 11 | dens evaginatus | symptomatic apical periodontitis |
| 10 | | female | 35 | | 11 | dens evaginatus | chronic apical abscess |
| 11 | | male | 35 | | 13 | dens evaginatus | asymptomatic apical periodontitis |
| 12 | | male | 11 | | 8 | trauma | symptomatic apical periodontitis |
| 13 | | female | 35 | | 10 | dens evaginatus | asymptomatic apical periodontitis |
| 14 | | male | 11 | | 12 | trauma | asymptomatic apical periodontitis |
| 15 | | female | 21 | | 10 | trauma | asymptomatic apical periodontitis |
| 16 | | female | 21 | | 8 | trauma | symptomatic apical periodontitis |
| 17 | | male | 45 | | 12 | dens evaginatus | chronic apical abscess |
| 18 | | male | 45 | | 12 | dens evaginatus | asymptomatic apical periodontitis |
| 19 | | female | 35 | | 10 | dens evaginatus | asymptomatic apical periodontitis |
| 20 | | male | 35 | | 10 | dens evaginatus | acute apical abscess |
| 21 | | female | 45 | | 9 | dens evaginatus | asymptomatic apical periodontitis |
| 22 | | female | 21 | | 9 | trauma | asymptomatic apical periodontitis |
| 23 | | female | 34 | | 10 | dens evaginatus | asymptomatic apical periodontitis |
| 24 | | male | 11 | | 9 | trauma | asymptomatic apical periodontitis |
| 25 | | female | 21 | | 14 | trauma | chronic apical abscess |
| 26 | | male | 35 | | 12 | dens evaginatus | asymptomatic apical periodontitis |
| 27 | | male | 11 | | 8 | trauma | symptomatic apical periodontitis |
| 28 | | male | 21 | | 8 | trauma | symptomatic apical periodontitis |
| 29 | | male | 35 | | 12 | dens evaginatus | asymptomatic apical periodontitis |
| 30 | | male | 15 | | 12 | dens evaginatus | asymptomatic apical periodontitis |
| 31 | | female | 35 | | 10 | dens evaginatus | asymptomatic apical periodontitis |
| 32 | | male | 45 | | 9 | dens evaginatus | symptomatic apical periodontitis |
| 33 | | male | 45 | | 10 | dens evaginatus | asymptomatic apical periodontitis |
| 34 | | female | 11 | | 9 | trauma | asymptomatic apical periodontitis |
| 35 | | male | 24 | | 9 | dens evaginatus | asymptomatic apical periodontitis |
| 36 | | male | 35 | | 12 | dens evaginatus | acute apical abscess |
| 37 | | female | 22 | | 12 | dens evaginatus | asymptomatic apical periodontitis |
| 38 | | male | 35 | | 11 | dens evaginatus | asymptomatic apical periodontitis |
|  | |  | 45 | | 11 | dens evaginatus | asymptomatic apical periodontitis |
| 39 | | female | 35 | | 11 | dens evaginatus | asymptomatic apical periodontitis |
| 40 | | female | 45 | | 9 | dens evaginatus | symptomatic apical periodontitis |
| 41 | | male | 21 | | 9 | trauma | asymptomatic apical periodontitis |
| 42 | | female | 35 | | 9 | dens evaginatus | chronic apical abscess |
| 43 | | male | 35 | | 10 | dens evaginatus | symptomatic apical periodontitis |
|  | |  | 45 | | 10 | dens evaginatus | asymptomatic apical periodontitis |
| 44 | | male | 21 | | 10 | trauma | asymptomatic apical periodontitis |
| 45 | | female | 45 | | 10 | dens evaginatus | asymptomatic apical periodontitis |
| 46 | | male | 45 | | 11 | dens evaginatus | asymptomatic apical periodontitis |
| 47 | | female | 44 | | 9 | dens evaginatus | symptomatic apical periodontitis |
| 48 | | female | 12 | | 13 | dens evaginatus | asymptomatic apical periodontitis |
| 49 | | male | 35 | | 11 | dens evaginatus | asymptomatic apical periodontitis |
| 50 | | female | 45 | | 9 | dens evaginatus | asymptomatic apical periodontitis |
| 51 | | female | 35 | | 12 | dens evaginatus | acute apical abscess |
| 52 | | male | 35 | | 12 | dens evaginatus | chronic apical abscess |
| 53 | | male | 21 | | 8 | trauma | asymptomatic apical periodontitis |
| 54 | | female | 21 | | 15 | trauma | asymptomatic apical periodontitis |
| 55 | | male | 11 | | 11 | trauma | chronic apical abscess |
| 56 | | female | 45 | | 8 | dens evaginatus | asymptomatic apical periodontitis |
| 57 | | female | 35 | | 12 | dens evaginatus | asymptomatic apical periodontitis |
| 58 | | female | 34 | | 9 | dens evaginatus | asymptomatic apical periodontitis |
| 59 | | female | 45 | | 10 | dens evaginatus | chronic apical abscess |
| 60 | | female | 21 | | 8 | trauma | asymptomatic apical periodontitis |
| 61 | | male | 45 | | 10 | dens evaginatus | symptomatic apical periodontitis |
| 62 | | female | 35 | | 10 | dens evaginatus | asymptomatic apical periodontitis |
| 63 | | male | 45 | | 11 | dens evaginatus | asymptomatic apical periodontitis |
| 64 | | male | 21 | | 9 | trauma | asymptomatic apical periodontitis |
| 65 | | female | 21 | | 7 | trauma | chronic apical abscess |
| 66 | | female | 25 | | 10 | dens evaginatus | symptomatic apical periodontitis |
| 67 | | male | 35 | | 10 | dens evaginatus | asymptomatic apical periodontitis |
| 68 | | female | 45 | | 16 | dens evaginatus | asymptomatic apical periodontitis |
| 69 | | male | 45 | | 13 | dens evaginatus | asymptomatic apical periodontitis |
| 70 | | male | 15 | | 10 | dens evaginatus | chronic apical abscess |
| 71 | | female | 45 | | 12 | dens evaginatus | symptomatic apical periodontitis |
| 72 | | female | 11 | | 9 | trauma | asymptomatic apical periodontitis |
| 73 | | female | 45 | | 10 | dens evaginatus | asymptomatic apical periodontitis |
| 74 | | male | 21 | | 7 | trauma | asymptomatic apical periodontitis |
| 75 | | female | 24 | | 10 | dens evaginatus | asymptomatic apical periodontitis |
| 76 | | male | 21 | | 9 | trauma | symptomatic apical periodontitis |
|  | | male | 11 | | 9 | trauma | asymptomatic apical periodontitis |
| 77 | | female | 21 | | 8 | trauma | asymptomatic apical periodontitis |
| 78 | | female | 35 | | 13 | dens evaginatus | chronic apical abscess |
| 79 | | male | 35 | | 13 | dens evaginatus | asymptomatic apical periodontitis |
| 80 | | female | 45 | | 9 | dens evaginatus | asymptomatic apical periodontitis |
| 81 | | male | 45 | | 13 | dens evaginatus | asymptomatic apical periodontitis |
| 82 | | female | 35 | | 9 | dens evaginatus | symptomatic apical periodontitis |
| 83 | | female | 15 | | 12 | dens evaginatus | chronic apical abscess |
| 84 | | female | 35 | | 10 | dens evaginatus | asymptomatic apical periodontitis |
| 85 | | female | 11 | | 8 | trauma | asymptomatic apical periodontitis |
|  | |  | 21 | | 8 | trauma | asymptomatic apical periodontitis |
| 86 | | male | 35 | | 9 | dens evaginatus | asymptomatic apical periodontitis |
| 87 | | female | 35 | | 12 | dens evaginatus | asymptomatic apical periodontitis |
| 88 | | male | 11 | | 8 | trauma | asymptomatic apical periodontitis |
|  | |  | 21 | | 8 | trauma | chronic apical abscess |
| 89 | | female | 15 | | 12 | dens evaginatus | asymptomatic apical periodontitis |
| 90 | | female | 45 | | 11 | dens evaginatus | asymptomatic apical periodontitis |
| 91 | | female | 11 | | 8 | trauma | asymptomatic apical periodontitis |
| 92 | | male | 45 | | 12 | dens evaginatus | asymptomatic apical periodontitis |
| 93 | | female | 35 | | 11 | dens evaginatus | chronic apical abscess |
| 94 | | female | 45 | | 12 | dens evaginatus | asymptomatic apical periodontitis |
| 95 | | female | 45 | | 11 | dens evaginatus | symptomatic apical periodontitis |
| 96 | | male | 35 | | 12 | dens evaginatus | asymptomatic apical periodontitis |
| 97 | | female | 11 | | 9 | trauma | asymptomatic apical periodontitis |
|  | |  | 35 | | 10 | dens evaginatus | asymptomatic apical periodontitis |
| 98 | | female | 35 | | 10 | dens evaginatus | asymptomatic apical periodontitis |
| 99 | | female | 45 | | 10 | dens evaginatus | chronic apical abscess |
| 100 | | male | 21 | | 9 | trauma | asymptomatic apical periodontitis |
| 101 | | female | 45 | | 10 | dens evaginatus | asymptomatic apical periodontitis |
| 102 | | male | 35 | | 10 | dens evaginatus | symptomatic apical periodontitis |
| 103 | | male | 45 | | 11 | dens evaginatus | asymptomatic apical periodontitis |
| 104 | | female | 21 | | 10 | trauma | asymptomatic apical periodontitis |
| 105 | | male | 21 | | 7 | trauma | chronic apical abscess |
| 106 | | female | 45 | | 9 | dens evaginatus | asymptomatic apical periodontitis |
| 107 | | female | 21 | | 8 | trauma | symptomatic apical periodontitis |
| 108 | | male | 21 | | 7 | trauma | asymptomatic apical periodontitis |
| 109 | | female | 15 | | 10 | dens evaginatus | acute apical abscess |
| 110 | | male | 45 | | 12 | dens evaginatus | asymptomatic apical periodontitis |
