# Supplementary material for: Preoperative factors analysis on root development after regenerative endodontic procedures: a retrospective study
Source: BMC Oral Health. 2022 Sep 4;22:374. doi: 10.1186/s12903-022-02412-x (PMC9442966; doi:10.1186/s12903-022-02412-x)
Supplement: Supplementary file 3 — Additional file 3. Preoperative measurement of root morphology by CBCT. [file 12903_2022_2412_MOESM3_ESM.docx]

| **Supplementary 3. Preoperative measurement of root morphology by CBCT** | | | | | | | | | | | | | | | |
| --- | --- | --- | --- | --- | --- | --- | --- | --- | --- | --- | --- | --- | --- | --- | --- |
| No. | Mesio-  distal apical foramen size (mm) | Bucco-  lingual apical foramen size (mm) | Averaged root length (mm) | Root wall thickness (mm) | | | | | | | | | | | |
|  |  |  |  | Buccal thickness at 4 mm from the CEJ | Buccal thickness at 6 mm from the CEJ | Buccal thickness at 8 mm from the CEJ | Lingual thickness at 4 mm from the CEJ | Lingual thickness at 6 mm from the CEJ | Lingual thickness at 8 mm from the CEJ | Mesial thickness at 4 mm from the CEJ | Mesial thickness at 6 mm from the CEJ | Mesial thickness at 8 mm from the CEJ | Distal thickness at 4 mm from the CEJ | Distal thickness at 6 mm from the CEJ | Distal thickness at 8 mm from the CEJ |
| 1 | 1.5 | 2.2 | 13.5 | 1.8 | 1.6 | 1.4 | 2.0 | 1.8 | 1.6 | 1.6 | 1.4 | 1.2 | 1.5 | 1.3 | 1.1 |
| 2 | 1.6 | 2.6 | 12.2 | 2.0 | 1.7 | 1.5 | 1.8 | 1.6 | 1.4 | 1.6 | 1.5 | 1.3 | 1.5 | 1.3 | 1.1 |
| 3 | 2.7 | 4.6 | 12.1 | 1.8 | 1.5 | 1.0 | 1.4 | 1.2 | 0.7 | 1.4 | 1.2 | 0.7 | 1.4 | 1.2 | 0.9 |
| 4 | 3.7 | 5.4 | 9.1 | 1.8 | 1.5 | 1.1 | 1.5 | 1.3 | 1.1 | 1.4 | 1.2 | 0.9 | 1.4 | 1.2 | 1.1 |
| 5 | 1.7 | 2.3 | 13.4 | 1.8 | 1.6 | 1.4 | 1.7 | 1.4 | 1.2 | 1.4 | 1.2 | 1.0 | 1.5 | 1.3 | 1.1 |
| 6 | 1.6 | 3.2 | 12.5 | 1.6 | 1.4 | 1.1 | 2.0 | 1.6 | 1.3 | 1.3 | 1.2 | 1.0 | 1.3 | 1.1 | 1.0 |
| 7 | 2.1 | 3.1 | 11.0 | 1.7 | 1.5 | 1.3 | 1.7 | 1.5 | 1.2 | 1.4 | 1.2 | 1.1 | 1.6 | 1.3 | 1.1 |
| 8 | 3.7 | 4.7 | 9.1 | 1.8 | 1.5 | 1.0 | 1.4 | 1.2 | 0.7 | 1.4 | 1.2 | 0.7 | 1.4 | 1.2 | 0.9 |
| 9 | 2.7 | 4.1 | 11.5 | 1.5 | 1.3 | 1.1 | 1.5 | 1.4 | 1.3 | 1.4 | 1.2 | 1.0 | 1.5 | 1.3 | 1.1 |
| 10 | 2.8 | 3.5 | 12.2 | 1.9 | 1.7 | 1.3 | 2.0 | 1.6 | 1.3 | 1.5 | 1.3 | 1.1 | 1.4 | 1.3 | 1.0 |
| 11 | 2.2 | 4.1 | 10.1 | 1.6 | 1.3 | 1.1 | 1.7 | 1.5 | 1.1 | 1.4 | 1.3 | 0.9 | 1.4 | 1.3 | 1.0 |
| 12 | 2.3 | 3.1 | 12.1 | 2.1 | 1.6 | 1.3 | 1.8 | 1.4 | 1.2 | 2.1 | 1.5 | 1.1 | 1.5 | 1.2 | 0.9 |
| 13 | 2.3 | 4.0 | 10.7 | 1.7 | 1.4 | 1.1 | 1.8 | 1.6 | 1.2 | 1.5 | 1.3 | 1.4 | 1.4 | 1.2 | 1.1 |
| 14 | 1.4 | 3.4 | 11.4 | 2.0 | 1.8 | 1.6 | 1.8 | 1.6 | 1.3 | 1.7 | 1.5 | 1.4 | 1.8 | 1.7 | 1.5 |
| 15 | 1.7 | 2.4 | 14.5 | 2.2 | 2.0 | 1.8 | 2.2 | 2.0 | 1.9 | 2.0 | 1.9 | 1.7 | 1.9 | 1.7 | 1.5 |
| 16 | 1.8 | 2.1 | 11.5 | 1.9 | 1.5 | 1.3 | 1.8 | 1.6 | 1.1 | 1.9 | 1.5 | 1.1 | 1.6 | 1.3 | 1.0 |
| 17 | 1.8 | 1.8 | 12.7 | 1.8 | 1.4 | 1.3 | 1.7 | 1.3 | 1.1 | 2.0 | 1.8 | 1.6 | 1.4 | 1.2 | 1.1 |
| 18 | 2.5 | 4.3 | 11.9 | 1.8 | 1.5 | 1.2 | 1.6 | 1.3 | 1.0 | 1.3 | 1.0 | 0.9 | 1.6 | 1.3 | 0.9 |
| 19 | 2.7 | 3.6 | 9.1 | 1.8 | 1.5 | 1.3 | 1.6 | 1.4 | 1.1 | 1.4 | 1.2 | 1.0 | 1.6 | 1.4 | 1.2 |
| 20 | 2.7 | 3.6 | 9.1 | 1.8 | 1.5 | 1.0 | 1.4 | 1.2 | 0.7 | 1.4 | 1.2 | 0.7 | 1.4 | 1.2 | 0.9 |
| 21 | 4.0 | 5.4 | 7.5 | 1.5 | 1.2 | 0.8 | 1.3 | 1.0 | 0.8 | 1.5 | 1.1 | 0.7 | 1.4 | 1.1 | 0.8 |
| 22 | 1.2 | 4.1 | 11.5 | 1.2 | 1.0 | 0.8 | 1.3 | 1.1 | 0.9 | 1.3 | 1.1 | 1.0 | 1.5 | 1.3 | 1.1 |
| 23 | 1.8 | 2.6 | 12.1 | 2.2 | 1.7 | 1.5 | 1.8 | 1.6 | 1.4 | 1.7 | 1.5 | 1.2 | 1.9 | 1.5 | 1.3 |
| 24 | 4.3 | 5.5 | 9.3 | 1.2 | 1.0 | 0.8 | 1.2 | 0.9 | 0.8 | 1.4 | 1.2 | 1.0 | 1.5 | 1.3 | 1.1 |
| 25 | 2.5 | 3.4 | 11.2 | 1.6 | 1.4 | 1.2 | 1.6 | 1.5 | 1.3 | 1.7 | 1.5 | 1.3 | 1.6 | 1.4 | 1.2 |
| 26 | 2.1 | 3.1 | 11.1 | 1.5 | 1.3 | 1.1 | 1.6 | 1.4 | 1.2 | 1.7 | 1.5 | 1.3 | 1.6 | 1.4 | 1.2 |
| 27 | 3.1 | 4.5 | 12.3 | 2.0 | 1.7 | 1.4 | 1.8 | 1.6 | 1.4 | 2.2 | 2.0 | 1.7 | 1.9 | 1.7 | 1.5 |
| 28 | 3.4 | 4.4 | 12.9 | 2.0 | 1.7 | 1.5 | 1.8 | 1.5 | 1.3 | 1.7 | 1.5 | 1.4 | 1.9 | 1.7 | 1.5 |
| 29 | 1.5 | 2.5 | 12.8 | 1.7 | 1.5 | 1.3 | 1.7 | 1.5 | 1.3 | 1.7 | 1.5 | 1.3 | 1.6 | 1.3 | 1.3 |
| 30 | 1.5 | 3.6 | 11.5 | 1.5 | 1.3 | 0.7 | 1.5 | 1.2 | 1.0 | 1.2 | 1.1 | 0.8 | 1.2 | 0.9 | 0.8 |
| 31 | 3.7 | 4.6 | 10.5 | 1.8 | 1.6 | 1.2 | 1.5 | 1.2 | 0.9 | 1.4 | 1.1 | 0.8 | 1.4 | 1.0 | 0.9 |
| 32 | 2.7 | 4.0 | 8.6 | 1.5 | 1.3 | 1.0 | 1.3 | 1.2 | 0.5 | 1.2 | 1.0 | 0.6 | 1.2 | 1.0 | 0.3 |
| 33 | 3.6 | 4.6 | 10.0 | 1.6 | 1.3 | 0.9 | 1.7 | 1.3 | 1.1 | 1.5 | 1.1 | 0.9 | 1.4 | 1.1 | 0.9 |
| 34 | 1.1 | 1.4 | 14.3 | 1.8 | 1.6 | 1.4 | 1.8 | 1.6 | 1.4 | 2.0 | 1.8 | 1.6 | 1.8 | 1.6 | 1.4 |
| 35 | 1.7 | 2.1 | 13.4 | 1.8 | 1.7 | 1.5 | 1.8 | 1.6 | 1.4 | 1.7 | 1.5 | 1.3 | 1.8 | 1.5 | 1.3 |
| 36 | 1.9 | 3.5 | 12.5 | 1.7 | 1.5 | 1.3 | 1.7 | 1.5 | 1.3 | 1.8 | 1.6 | 1.4 | 1.6 | 1.3 | 1.2 |
| 37 | 4.3 | 6.0 | 8.7 | 2.0 | 1.0 | 0.4 | 2.3 | 1.4 | 0.2 | 1.8 | 1.4 | 0.6 | 2.1 | 1.8 | 1.2 |
| 38 | 2.2 | 2.5 | 12.5 | 1.9 | 1.7 | 1.3 | 2.0 | 1.6 | 1.3 | 1.5 | 1.3 | 1.1 | 1.4 | 1.3 | 1.0 |
| 39 | 2.3 | 4.0 | 10.9 | 2.0 | 1.8 | 1.2 | 1.8 | 1.5 | 1.0 | 1.2 | 0.9 | 0.7 | 1.7 | 1.3 | 0.8 |
| 40 | 2.4 | 3.3 | 10.3 | 1.8 | 1.5 | 1.4 | 1.7 | 1.5 | 1.3 | 1.6 | 1.4 | 0.1 | 1.7 | 1.5 | 1.2 |
| 41 | 1.7 | 2.6 | 11.8 | 1.4 | 1.1 | 0.4 | 1.5 | 1.1 | 0.8 | 1.1 | 0.8 | 0.7 | 1.4 | 1.0 | 0.6 |
| 42 | 3.5 | 3.1 | 13.1 | 1.7 | 1.5 | 1.3 | 1.7 | 1.5 | 1.3 | 1.7 | 1.5 | 1.3 | 1.8 | 1.6 | 1.4 |
| 43 | 2.3 | 4.4 | 11.6 | 1.5 | 1.3 | 1.0 | 1.7 | 1.5 | 1.0 | 1.5 | 1.1 | 0.8 | 1.3 | 1.2 | 0.9 |
| 44 | 1.2 | 0 | 12.1 | 2.0 | 1.7 | 1.6 | 1.4 | 1.3 | 1.3 | 1.3 | 1.3 | 1.1 | 1.4 | 1.4 | 1.2 |
| 45 | 1.5 | 1.9 | 12.1 | 1.7 | 1.4 | 1.0 | 1.8 | 1.5 | 1.4 | 1.3 | 1.1 | 0.9 | 1.3 | 1.1 | 1.0 |
| 46 | 1.8 | 2.0 | 12.8 | 1.9 | 1.7 | 1.5 | 1.8 | 1.6 | 1.4 | 2.0 | 1.8 | 1.6 | 1.8 | 1.7 | 1.3 |
| 47 | 2.6 | 4.6 | 10.2 | 2.2 | 1.7 | 1.1 | 2.0 | 1.6 | 1.2 | 1.6 | 1.5 | 1.4 | 1.4 | 1.2 | 1.1 |
| 48 | 2.5 | 3.7 | 11.2 | 1.7 | 1.6 | 1.3 | 1.6 | 1.5 | 1.1 | 1.2 | 1.2 | 1.0 | 1.3 | 1.2 | 1.0 |
| 49 | 2.0 | 2.8 | 12.2 | 1.7 | 1.5 | 1.3 | 1.7 | 1.6 | 1.4 | 1.8 | 1.6 | 1.4 | 1.7 | 1.5 | 1.3 |
| 50 | 2.2 | 1.9 | 13.1 | 1.7 | 1.3 | 1.2 | 1.6 | 1.5 | 1.2 | 1.5 | 1.1 | 1.0 | 1.5 | 1.4 | 1.1 |
| 51 | 1.5 | 3.2 | 11.7 | 1.7 | 1.5 | 1.4 | 1.7 | 1.1 | 1.1 | 1.1 | 1.1 | 1.0 | 1.1 | 1.0 | 0.9 |
| 52 | 3.7 | 4.4 | 10.9 | 1.7 | 1.3 | 1.1 | 1.5 | 1.2 | 0.9 | 1.4 | 1.3 | 0.8 | 1.4 | 1.0 | 0.7 |
| 53 | 1.5 | 2.3 | 11.0 | 1.7 | 1.4 | 1.3 | 1.6 | 1.4 | 1.2 | 1.3 | 1.2 | 1.0 | 1.4 | 1.3 | 1.0 |
| 54 | 2.2 | 2.5 | 10.5 | 1.9 | 1.7 | 1.5 | 2.0 | 1.6 | 1.3 | 1.5 | 1.3 | 1.1 | 1.4 | 1.3 | 1.0 |
| 55 | 1.8 | 3.3 | 12.6 | 2.1 | 1.9 | 1.7 | 1.8 | 1.7 | 1.7 | 1.7 | 1.4 | 1.2 | 2.0 | 1.7 | 1.5 |
| 56 | 3.8 | 3.9 | 12.9 | 1.8 | 1.6 | 1.4 | 1.9 | 1.6 | 1.4 | 1.7 | 1.5 | 1.3 | 1.8 | 1.6 | 1.4 |
| 57 | 2.1 | 3.4 | 14.9 | 2.2 | 2.0 | 1.8 | 2.0 | 1.8 | 1.6 | 2.3 | 1.7 | 1.3 | 1.8 | 1.6 | 1.3 |
| 58 | 1.3 | 2.4 | 12.8 | 1.5 | 1.6 | 1.6 | 1.8 | 1.3 | 0.9 | 1.4 | 1.4 | 1.3 | 1.4 | 1.2 | 1.1 |
| 59 | 2.0 | 3.3 | 9.1 | 1.4 | 1.2 | 1.1 | 1.6 | 1.2 | 1.1 | 1.4 | 1.3 | 1.1 | 1.4 | 1.2 | 1.1 |
| 60 | 2.0 | 2.0 | 12.5 | 1.7 | 1.5 | 1.3 | 1.7 | 1.5 | 1.4 | 1.8 | 1.6 | 1.4 | 1.7 | 1.5 | 1.4 |
| 61 | 1.8 | 2.6 | 13.1 | 1.8 | 1.4 | 0.9 | 1.7 | 1.6 | 1.4 | 1.4 | 1.1 | 0.9 | 1.6 | 1.3 | 1.3 |
| 62 | 1.8 | 4.9 | 14.9 | 1.6 | 1.3 | 1.9 | 1.5 | 1.1 | 0.9 | 1.5 | 1.4 | 1.1 | 1.6 | 1.2 | 0.8 |
| 63 | 1.9 | 3.5 | 12.3 | 1.5 | 1.5 | 1.1 | 1.7 | 1.4 | 1.3 | 1.4 | 1.2 | 0.7 | 1.5 | 1.2 | 1.2 |
| 64 | 1.9 | 0 | 12.0 | 1.7 | 1.5 | 1.3 | 1.7 | 1.5 | 1.3 | 1.6 | 1.3 | 1.2 | 1.6 | 1.4 | 1.2 |
| 65 | 1.3 | 2.0 | 13.1 | 1.7 | 1.5 | 1.3 | 1.7 | 1.5 | 1.3 | 1.8 | 1.6 | 1.4 | 1.7 | 1.5 | 1.3 |
| 66 | 1.4 | 3.7 | 13.8 | 2.0 | 1.5 | 1.2 | 1.8 | 1.6 | 1.1 | 1.8 | 1.4 | 1.0 | 1.5 | 1.2 | 0.9 |
| 67 | 1.8 | 2.9 | 12.2 | 1.5 | 1.3 | 1.1 | 1.5 | 1.3 | 1.1 | 1.7 | 1.4 | 1.1 | 1.7 | 1.5 | 1.3 |
| 68 | 2.0 | 3.6 | 11.8 | 1.7 | 1.5 | 1.3 | 1.7 | 1.5 | 1.3 | 1.8 | 1.5 | 1.3 | 1.6 | 1.4 | 1.2 |
| 69 | 3.2 | 3.2 | 8.8 | 1.6 | 1.4 | 1.1 | 1.5 | 1.4 | 0.8 | 1.5 | 1.4 | 0.8 | 1.5 | 1.1 | 0.8 |
| 70 | 2.1 | 3.4 | 10.0 | 1.7 | 1.5 | 1 | 1.6 | 1.2 | 1 | 1.4 | 1.2 | 1.1 | 1.6 | 1.5 | 1.1 |
| 71 | 2.7 | 3.1 | 11.5 | 1.7 | 1.3 | 1.1 | 1.7 | 1.5 | 1.3 | 1.4 | 1.2 | 1.0 | 1.5 | 1.3 | 1.1 |
| 72 | 2.7 | 3.7 | 11.0 | 1.6 | 1.4 | 1.2 | 1.7 | 1.5 | 1.3 | 1.7 | 1.5 | 1.3 | 1.7 | 1.4 | 1.2 |
| 73 | 2.4 | 1.6 | 9.3 | 1.7 | 1.5 | 1.3 | 1.6 | 1.4 | 1.2 | 1.7 | 1.5 | 1.3 | 1.6 | 1.5 | 1.3 |
| 74 | 1.0 | 1.2 | 13.9 | 2.0 | 1.8 | 1.7 | 2.0 | 1.8 | 1.7 | 2.0 | 1.8 | 1.7 | 2.1 | 1.9 | 1.7 |
| 75 | 1.8 | 4.6 | 10.9 | 1.4 | 1.3 | 1.3 | 1.7 | 1.4 | 1.1 | 1.4 | 1.3 | 1.2 | 1.5 | 1.4 | 1.0 |
| 76 | 1.5 | 1.9 | 15.3 | 2.0 | 1.8 | 1.6 | 1.9 | 1.7 | 1.5 | 1.7 | 1.5 | 1.3 | 1.7 | 1.5 | 1.3 |
| 77 | 1.7 | 3.6 | 11.2 | 1.3 | 1.4 | 1.1 | 1.7 | 1.7 | 1.2 | 1.6 | 1.4 | 1.3 | 1.5 | 1.2 | 1.1 |
| 78 | 2.3 | 4.4 | 12.6 | 1.4 | 0.7 | 0.3 | 1.3 | 0.6 | 0.3 | 1.7 | 1.5 | 1.3 | 1.6 | 1.4 | 1.2 |
| 79 | 2.7 | 3.6 | 13.0 | 1.9 | 1.7 | 1.5 | 1.7 | 1.5 | 1.3 | 1.5 | 1.3 | 1.1 | 1.5 | 1.3 | 1.1 |
| 80 | 1.3 | 1.7 | 15.0 | 1.9 | 1.7 | 1.5 | 1.8 | 1.6 | 1.4 | 1.8 | 1.6 | 1.4 | 1.8 | 1.6 | 1.4 |
| 81 | 2.0 | 3.3 | 10.5 | 1.8 | 1.5 | 1.2 | 1.6 | 1.4 | 1.1 | 1.5 | 1.3 | 1.0 | 1.4 | 1.1 | 0.8 |
| 82 | 2.1 | 3.2 | 10.3 | 1.9 | 1.7 | 1.5 | 1.8 | 1.6 | 1.4 | 1.7 | 1.5 | 1.3 | 1.7 | 1.5 | 1.3 |
| 83 | 1.9 | 3.5 | 12.3 | 1.5 | 1.5 | 1.1 | 1.7 | 1.4 | 1.3 | 1.4 | 1.2 | 0.7 | 1.5 | 1.2 | 1.2 |
| 84 | 1.9 | 2.1 | 12.0 | 1.7 | 1.5 | 1.3 | 1.7 | 1.5 | 1.3 | 1.6 | 1.3 | 1.2 | 1.6 | 1.4 | 1.2 |
| 85 | 2.5 | 3.0 | 8.5 | 1.3 | 1.0 | 0.7 | 1.5 | 1.2 | 0.8 | 1.5 | 1.4 | 1.2 | 1.6 | 1.4 | 1.2 |
| 86 | 1.4 | 2.6 | 10.5 | 1.7 | 1.5 | 1.3 | 1.8 | 1.6 | 1.4 | 1.6 | 1.4 | 1.2 | 1.7 | 1.4 | 1.2 |
| 87 | 2.9 | 5.7 | 9.6 | 1.4 | 1.0 | 0.6 | 1.5 | 1.2 | 0.9 | 1.6 | 1.4 | 1.2 | 1.7 | 1.5 | 1.3 |
| 88 | 2.8 | 2.8 | 10.8 | 1.8 | 1.5 | 1.1 | 1.8 | 1.4 | 1.2 | 1.6 | 1.4 | 1.2 | 1.5 | 1.3 | 1.1 |
| 89 | 1.6 | 3.0 | 14.4 | 2.0 | 1.8 | 1.6 | 1.9 | 1.7 | 1.5 | 1.8 | 1.6 | 1.5 | 1.8 | 1.6 | 1.4 |
| 90 | 3.8 | 5.5 | 9.1 | 1.7 | 1.2 | 0.4 | 1.6 | 1.1 | 0.5 | 1.6 | 1.2 | 0.8 | 1.5 | 1.2 | 0.8 |
| 91 | 3.1 | 2.6 | 10.2 | 1.7 | 1.2 | 0.6 | 1.6 | 1.2 | 0.7 | 1.6 | 1.4 | 1.2 | 1.7 | 1.5 | 1.3 |
| 92 | 1.6 | 2.9 | 13.3 | 1.7 | 1.5 | 1.3 | 1.6 | 1.4 | 1.2 | 1.9 | 1.7 | 1.5 | 1.9 | 1.7 | 1.5 |
| 93 | 2.8 | 3.1 | 12.9 | 1.5 | 1.3 | 1.2 | 1.5 | 1.3 | 1.7 | 1.7 | 1.4 | 1.1 | 1.7 | 1.5 | 1.3 |
| 94 | 2.3 | 3.9 | 12.2 | 1.8 | 1.6 | 1.4 | 1.8 | 1.6 | 1.4 | 1.7 | 1.5 | 1.3 | 1.7 | 1.5 | 1.3 |
| 95 | 3.0 | 4.5 | 10.7 | 1.8 | 1.6 | 1.4 | 1.7 | 1.5 | 1.4 | 1.7 | 1.5 | 1.3 | 1.7 | 1.5 | 1.3 |
| 96 | 1.6 | 2.6 | 12.6 | 1.7 | 1.5 | 1.3 | 1.7 | 1.5 | 1.3 | 1.5 | 1.3 | 1.1 | 1.5 | 1.3 | 1.2 |
| 97 | 1.5 | 1.9 | 12.3 | 1.8 | 1.6 | 1.4 | 1.7 | 1.4 | 1.2 | 1.7 | 1.5 | 1.3 | 1.7 | 1.5 | 1.3 |
| 98 | 3.5 | 4.6 | 8.9 | 1.7 | 1.2 | 0.9 | 1.6 | 1.2 | 0.9 | 1.6 | 1.2 | 0.8 | 1.5 | 1.2 | 1.0 |
| 99 | 2.2 | 2.2 | 13.8 | 1.7 | 1.5 | 1.3 | 1.8 | 1.6 | 1.4 | 1.7 | 1.5 | 1.3 | 1.7 | 1.5 | 1.3 |
| 100 | 1.5 | 2.2 | 11.8 | 1.6 | 1.4 | 1.2 | 1.6 | 1.4 | 1.2 | 1.6 | 1.4 | 1.2 | 1.8 | 1.6 | 1.4 |
| 101 | 2.1 | 2.4 | 12.7 | 1.8 | 1.6 | 1.4 | 1.7 | 1.5 | 1.3 | 1.7 | 1.5 | 1.3 | 1.7 | 1.4 | 1.1 |
| 102 | 2.0 | 2.2 | 13.6 | 1.8 | 1.6 | 1.4 | 1.8 | 1.6 | 1.4 | 1.8 | 1.6 | 1.4 | 1.8 | 1.6 | 1.4 |
| 103 | 2.0 | 3.3 | 9.1 | 1.4 | 1.2 | 1.1 | 1.6 | 1.2 | 1.1 | 1.4 | 1.3 | 1.1 | 1.4 | 1.2 | 1.1 |
| 104 | 1.8 | 2.0 | 12.4 | 1.7 | 1.5 | 1.3 | 1.7 | 1.5 | 1.4 | 1.8 | 1.6 | 1.4 | 1.7 | 1.5 | 1.4 |
| 105 | 1.8 | 2.6 | 13.1 | 1.8 | 1.4 | 0.9 | 1.7 | 1.6 | 1.4 | 1.4 | 1.1 | 0.9 | 1.6 | 1.3 | 1.3 |
| 106 | 1.8 | 4.9 | 14.9 | 1.6 | 1.3 | 1.9 | 1.5 | 1.1 | 0.9 | 1.5 | 1.4 | 1.1 | 1.6 | 1.2 | 0.8 |
| 107 | 2.3 | 3.7 | 8.4 | 1.7 | 1.3 | 1.1 | 1.5 | 1.2 | 0.9 | 1.7 | 1.5 | 1.3 | 1.5 | 1.4 | 1.2 |
| 108 | 1.9 | 2.8 | 13.1 | 1.8 | 1.6 | 1.4 | 1.7 | 1.5 | 1.3 | 1.8 | 1.6 | 1.4 | 1.7 | 1.5 | 1.3 |
| 109 | 1.3 | 2.0 | 13.1 | 1.7 | 1.5 | 1.3 | 1.7 | 1.5 | 1.3 | 1.8 | 1.6 | 1.4 | 1.7 | 1.5 | 1.3 |
| 110 | 1.4 | 2.7 | 10.8 | 2.0 | 1.5 | 1.2 | 1.8 | 1.6 | 1.1 | 1.8 | 1.4 | 1.0 | 1.5 | 1.2 | 0.9 |
| 111 | 1.8 | 2.9 | 12.2 | 1.5 | 1.3 | 1.1 | 1.5 | 1.3 | 1.1 | 1.7 | 1.4 | 1.1 | 1.7 | 1.5 | 1.3 |
| 112 | 1.3 | 2.1 | 13.1 | 1.7 | 1.5 | 1.0 | 1.7 | 1.5 | 1.3 | 1.8 | 1.6 | 1.4 | 1.7 | 1.5 | 1.3 |
| 113 | 1.9 | 3.8 | 13.2 | 2.1 | 1.5 | 1.1 | 1.8 | 1.6 | 1.5 | 1.8 | 1.4 | 1.0 | 1.5 | 1.2 | 0.10 |
| 114 | 2.8 | 3.1 | 12.3 | 1.5 | 1.3 | 1.2 | 1.5 | 1.3 | 1.7 | 1.7 | 1.4 | 1.1 | 1.7 | 1.5 | 1.3 |
| 115 | 2.7 | 3.7 | 11.0 | 1.6 | 1.4 | 1.2 | 1.7 | 1.5 | 1.3 | 1.7 | 1.5 | 1.3 | 1.7 | 1.4 | 1.2 |
| 116 | 2.4 | 2.6 | 9.3 | 1.7 | 1.5 | 1.3 | 1.6 | 1.4 | 1.2 | 1.7 | 1.5 | 1.3 | 1.6 | 1.5 | 1.3 |
